# Correction: The Burr distribution as a model for the delay between key events in an individual’s infection history

**DOI:** 10.1371/journal.pcbi.1014163

**Published:** 2026-04-06

**Authors:** Nyall Jamieson, Christiana Charalambous, David M. Schultz, Ian Hall

In the A probability-based approach subsection of the Materials and methods, there are errors in the first two paragraphs. The correct paragraphs are:

A continuous-time mathematical model can be built considering the hazard rate of symptom-onset occurrence. We first consider the option of using an exponential survival model with time-varying hazard. Define N(t) as the population of individuals who are infected, but are not yet symptomatic at time t, and Q(t) as the population of individuals who are symptomatic at time t with $N(\text{0})=N_{\text{0}},\;Q(\text{0})=\text{0}$ and $Q(t)+N(t)=N_{\text{0}},\;\forall t\in R^{+}.$ Next, assume that a hazard rate function λ(t) describes the risk that a not-yet-symptomatic individual will start to experience symptoms at a point in time t, given that they have not already succumb to symptoms by time t. Then 1−δtλ(t) will be the probability that the individual will remain asymptomatic within a small interval (t,t+δt), where in this context δt  represents a small time increment in time. Hence ${\left(\text{1}-\delta t\lambda (t)\right)}^{N(t)}$ is the probability that nobody who is not-yet-symptomatic will start experiencing symptoms within a small increment δt  from t . Following this, define $\delta Q(t)=\text{1}-{\left(\text{1}-\delta t\lambda (t)\right)}^{N(t)}$ to be the probability that there is at least one individual who starts to experience new symptoms in a small increment δt  from t . For the small increment δt, we may approximate ${\left(\text{1}-\delta t\lambda (t)\right)}^{N(t)}\approx e^{-\lambda (t)N(t)\delta t}$. Therefore, the probability of any new symptom onset can be written as $\delta Q(t)=\text{1}-e^{-\lambda (t)N(t)\delta t}$. Using a Taylor expansion on the exponential term, dividing by δt , and taking the limit as δt→0  changes this probability to a rate as follows:


$$\frac{dQ(t)}{dt}=\lambda (t)N(t)=\lambda (t)\left(N_{\text{0}}-Q(t)\right)$$

This approach leads to a separable ordinary differential equation analogous to the cumulative distribution of the exponential distribution with a time-varying rate parameter. It can be deduced that $F(t)=\text{1}-\text{exp}\left(-\int_{\text{0}}^{t}\lambda (\tau )d\tau \right)$ and that $\int_{\text{0}}^{t}\lambda (\tau )d\tau$ is the accumulated hazard. The scenario discussed here can be considered from an inhomogeneous Poisson-process perspective, and the results of the hazard are identical to the inhomogeneous exponentially distributed model. It can be noted here that if λ(t) is constant that this would lead to the exponential distribution and if $\lambda (t)\propto t^{a}$ for some constant a  this would suggest the incubation period is a Weibull distributed random variable. The Erlang distribution arises by assuming the incubation period is the sum of a number of stages of constant length λ.

In the General derived Burr distribution subsection of the Materials and methods, there is an error in the first sentence of the first paragraph. The correct sentence is: In [3], g(t) has a physical interpretation; the function tends to the rate of symptom onset λ(t) in individuals at a time t as t increases.

In Table 1, the parameter range for the type III distribution and type XII distribution. Please see the correct Table 1 here.

**Table 1:** The Burr distributions valid over (0,∞) and previously trialled distributions with their corresponding p.d.f. and c.d.f.

Distribution	p.d.f	c.d.f	Parameter range
Log-normal	$\frac{\text{1}}{t\sigma \sqrt{\text{2}\pi }}e^{-\frac{{\left(\text{log}(t)-\mu \right)}^{\text{2}}}{\text{2}\sigma^{\text{2}}}}$	$\frac{\text{1}}{\text{2}}\left[\text{1}+\text{erf}\left(\frac{\text{log}(t)-\mu }{\sigma \sqrt{\text{2}}}\right)\right]$	μ∈R,σ>0
Weibull	$\frac{k}{\lambda }{\left(\frac{t}{\lambda }\right)}^{k-\text{1}}e^{-{\left(\frac{t}{\lambda }\right)}^{k}}$	$\text{1}-e^{-{\left(\frac{t}{\lambda }\right)}^{k}}$	k,λ>0
Gamma	$\frac{\beta^{-\alpha }}{\Gamma (\alpha )}t^{\alpha -\text{1}}e^{-\frac{t}{\beta }}$	$\frac{\text{1}}{\Gamma (\alpha )}\gamma (\alpha ,\;\beta t)$	α,β>0
Type III	$\frac{\alpha \beta }{t}{\left(\frac{t}{\gamma }\right)}^{-\alpha }{\left(\text{1}+{\left(\frac{t}{\gamma }\right)}^{-\alpha }\right)}^{-\beta -\text{1}}$	${\left(\text{1}+{\left(\frac{t}{\gamma }\right)}^{-\alpha }\right)}^{-\beta }$	α,β,γ>0
Type X	$\frac{\text{2}\alpha t\;}{\gamma^{\text{2}}}e^{-{\left(\frac{t}{\gamma }\right)}^{\text{2}\;}}{\left(\text{1}-e^{-{\left(\frac{t}{\gamma }\right)}^{\text{2}}}\right)}^{\alpha -\text{1}}$	${\left(\text{1}-e^{-{\left(\frac{t}{\gamma }\right)}^{\text{2}}}\right)}^{\alpha }$	α,γ>0
Type XII	$\frac{\alpha (\beta -\text{1})}{T}\left({\text{2}}^{\frac{\text{1}}{\beta -\text{1}}}-\text{1}\right){\left(\text{1}+\left({\text{2}}^{\frac{\text{1}}{\beta -\text{1}}}-\text{1}\right){\left(\frac{t}{T}\right)}^{\alpha }\right)}^{-\beta }{\left(\frac{t}{T}\right)}^{\alpha -\text{1}}$	$\text{1}-{\left(\text{1}+\left({\text{2}}^{\frac{\text{1}}{\beta -\text{1}}}-\text{1}\right){\left(\frac{t}{T}\right)}^{\alpha }\right)}^{\text{1}-\beta }$	α,T>0,β>1
Derived	$\frac{\left(\frac{t}{\beta }+\alpha \right){\left(\frac{T}{t}\right)}^{\alpha }e^{\frac{\left(T-t\right)}{\beta }}}{t{\left(\text{1}+{\left(\frac{T}{t}\right)}^{\alpha }e^{\frac{\left(T-t\right)}{\beta }}\right)}^{\text{2}}}$	$\frac{\text{1}}{\text{1}+{\left(\frac{T}{t}\right)}^{\alpha }e^{\frac{T-t}{\beta }}}$	α,β,T>0
